# Targeting STAT3 Abrogates Tim-3 Upregulation of Adaptive Resistance to PD-1 Blockade on Regulatory T Cells of Melanoma

**DOI:** 10.3389/fimmu.2021.654749

**Published:** 2021-04-15

**Authors:** Lili Huang, Yu Xu, Juemin Fang, Weixing Liu, Jianhua Chen, Zhuqing Liu, Qing Xu

**Affiliations:** ^1^Department of Oncology, Shanghai Tenth People’s Hospital, Tongji University School of Medicine, Shanghai, China; ^2^Tongji University Cancer Center, Shanghai, China; ^3^Department of Musculoskeletal Oncology, Fudan University Shanghai Cancer Center, Shanghai, China; ^4^Department of Oncology, Shanghai Medical College, Fudan University, Shanghai, China

**Keywords:** regulatory T cells, Tim-3, melanoma, anti-PD-1 therapy, STAT3

## Abstract

**Background:**

Less than 20% of melanoma patients respond to programmed cell death-1 (PD-1) blockade immunotherapies. Thus, it is crucial to understand the dynamic changes in the tumor microenvironment (TME) after PD-1 blockade, for developing immunotherapy efficacy.

**Methods:**

A genomic analysis was conducted by The Cancer Genome Atlas (TCGA) datasets and web platform TIMER2.0 datasets. Pathway enrichment analysis was performed using the Kyoto Encyclopedia of Genes and Genomes (KEGG) pathway. Peripheral blood mononuclear cells (PBMCs), regulatory T (Treg) cells, and B16-F10 melanoma mice were used as models. The cellular and molecular characteristics and mechanisms of Treg cells in melanoma were assessed by performing gene expression studies, immunohistochemistry, RNA sequencing, and flow cytometry.

**Results:**

Here, we evaluate the countenance of T cell immunoglobulin and mucin-domain containing-3 (Tim-3), and various immunosuppressive factors within tumor-infiltrated Treg cells after treatment with anti-PD-1 or the indicator transduction and activator of transcription 3 (STAT3) inhibitors. Increased expression of Tim-3 is markedly observed within the tissues of the PD-1 blockade resistance of melanoma patients. Targeting STAT3 significantly boosts the response of resistant-PD-1 therapy within the melanoma mouse model. Mechanistically, the manifestation of STAT3 decreases the expression of Tim-3 and various cytokines in the purified Treg cells from individual PBMCs and the murine melanoma model, limiting the immunosuppression of Treg cells.

**Conclusions:**

Our findings indicate that Tim-3 expression on Treg cells within the TME is STAT3-dependent, providing support to STAT3 as a target and enhancing the immunotherapy for patients suffering from melanoma.

## Background

Recently, designed programmed cell death-1 (PD-1) blocking antibodies have been found to produce excellent effects concerning the medication of numerous tumors, containing melanoma, non-small-cell lung cell cancer, and bladder cancer (1–3). However, patients who receive nivolumab show low responses (4, 5). Thus, it is essential to comprehend the vibrant alterations of adaptive resistance within the tumor microenvironment (TME) after PD-1 blockade and explore novel intervention targets as a possible therapy for improving the efficacy of immunotherapy.

With the development of the study of the TME, innovative and synergistic combination therapies were found to have excellent efficacy in melanoma. While blocking one more immunomodulatory pathway is still needed to explore the eradication of advanced melanomas (6). One prominent strategy includes blocking dual immune harrier receptors in T cells by targeting both the inhibitory T cell immunoglobulin and mucin-domain containing-3 (Tim-3) and PD-1 (7). Tim-3 is considered the checkpoint receptor mediating T cell tiredness in the immune microenvironment (8, 9). Tumor-associated Treg cells, which emphasize inhibitory checkpoint receptor Tim-3, comprise a particular subset of tissue Treg cells by promoting tumor progression in mouse or human melanoma tissues by binding to galectin-9 which is expressed in melanoma cells (10, 11). Moreover, methods targeting T cells *via* the PD-1 immune checkpoint pathway resulted in increased Tim-3 proportion (12, 13). The previous research showed that effective anti-PD-1 treatment could decrease Tim-3 expression on the Treg cells of head and neck squamous cell carcinoma (HNSCC) tumors (14). However, the dynamic changes of Tim-3 on Treg cells in anti-PD-1 nonresponse melanoma patients, the potential cross-talk of PD-1 and Tim-3 in tumor-infiltrating lymphocytes (TILs), and the mechanism of the regulation of the two checkpoint receptors above have not been elucidated.

Indicator transduction and activator of transcription 3 (STAT3) constitutively mediate tumor proliferation, progression and metastasis, and immunity in the TME (15, 16). STAT3 activation in adaptive immune cells results in inhibiting immune regulation and immunosuppressive factors secretion (17). Moreover, STAT3 acts as a co-transcription pathway for forehead box P3 (Foxp3) and a mediator for other STAT3-associated targets, including interleukin-10 (IL-10), in Treg cells (18). Furthermore, STAT3 has been identified to promote the articulation of Foxp3 within CD4^+^CD25^+^ Treg cells in the TME, enhancing immune suppressive function (19, 20). A STAT3 inhibitor WP1066 could produce an excellent antitumor response due to the inhibition of the proliferation of Treg cells (21). Furthermore, STAT3 can directly control the promotor of converting development component in Treg cells of T cell-precise SOCS3-inadequate mice (22). As to whether and how the STAT3 pathway regulates the inhibitory checkpoint receptor Tim-3 in the TME is still unknown.

In this study, we observed that higher expression of Tim-3 on Treg cells is associated with the resistance of melanoma patients treated with anti-PD-1. Moreover, using Stattic to target the STAT3 pathway, we observed Tim-3 downregulation in *in vitro* phenotype and function assays and an *in vivo* melanoma model. Interestingly, depletion of STAT3 in the preclinical model dampened the tumor growth and Treg cells recruitment and increased the CD8^+^/CD4^+^ ratio in melanoma. Furthermore, synergistic combination therapies with Stattic impairs the responses to anti-PD-1 melanoma. Our findings reveal that combining STAT3 downregulation and anti-PD-1 could be used as a potential medicinal target for diseased persons.

## Materials and Methods

### The Cancer Genome Atlas (TCGA) Analysis

The melanoma dataset was transferred from the TCGA program using cBioportal for Cancer Genomics (www.cbioportal.org). The gene expression of PDCD1, HACVR2, and STAT3 was evaluated using Spearman’s rank correlation coefficient using the publicly available TCGA dataset. The patients’ clinical information along with the mRNA expression data (RNA Seq V2 RSEM) were acquired from cBioportal for Cancer Genomics (www.cbioportal.org). The association between immune infiltrates and genetic and clinical features were estimated by web platform TIMER2.0 datasets (http://timer.cistrome.org/). Kaplan-Meier curve analysis and log-rank tests were performed to observe the survival rate of melanoma patients using web platform TIMER2.0 datasets (http://timer.cistrome.org/).

### Correlation and Pathway Enrichment Analysis

The gene expression of PDCD1, HACVR2, and TIL Treg cells was performed in primary and metastasis melanoma using the *t-test*. Patients from TCGA were divided into two quartiles according to the expression of PDCD1 stated in a divergent way. Genes were discovered that corresponded to the criteria of *p* < 0.05, fold change > 1.5. The top significantly differentially expressed genes were determined in the PD-1 high representation and PD-1 low representation groups by the *p* values (n=472). To cluster these genes in the pathway of melanoma, we uploaded the related genes to the Database for Annotation, Visualization, and Integrated Discovery (DAVID) functional annotation analysis website (https://david.ncifcrf.gov/). We selected the top 30 pathways in the Kyoto Encyclopedia of Genes and Genomes (KEGG) pathways analysis.

### Reagents

The anti-human PD-1 antibody (used at a 10 μg/mL concentration) was obtained from Merck & Co., Inc. (New Jersey, USA). In VivoM Ab anti-mouse PD-1 (used at a 10 μg/mL concentration) and In VivoM Ab anti-mouse IgG2a were purchased from Bioxcell (New Hampshire, USA). STAT3 inhibitor Stattic (used at a 5 μM concentration) was purchased from MedChemExpress (New Jersey, USA). Stattic was dissolved in DMSO. Recombinant human IL-2 (used at a 200 IU/mL concentration) was obtained from Thermo Fisher Scientific (Waltham, USA) and solubilized in PBS with 1% BSA. Ultra-LEAF™ Purified anti-human CD3 antibody (used at 1 µg/mL concentration, clone: OKT3), Ultra-LEAF™ Purified anti-human CD28 antibody (1 µg/mL concentration, clone: 28.2), and cell activation cocktail (with Brefeldin A) were purchased from Biolegend (San Diego, USA). Fixable viability Dye eFluor™ 780 was obtained from Thermo Fisher Scientific (Waltham, USA) and solubilized in DMSO.

### Cell Culture

Melanoma cell lines B16-F10 were purchased from the American Type Culture Collection (ATCC). Cells were refined at 37°C with 5% CO2 and preserved within Dulbecco’s Modified Eagle Medium (DMEM) (Gibco, USA) with 10% fetal bovine serum (FBS) (Gibco, USA) and 1% penicillin/streptomycin (Gibco, USA). Peripheral blood mononuclear cells (PBMCs) and purified Treg cells were cultured in 1640 medium (10% FBS) with 1% penicillin/streptomycin after the plate was coated with anti-CD3, anti-CD28, and IL-2.

### Patients and Samples

Human specimens were collected with the approval of Tongji University. Blood samples were taken from healthy donors, centrifuged (2,000 rpm for 20 min at 4°C), and accumulated for flow cytometry analysis. Tissue samples were obtained and isolated from melanoma patients for flow cytometry and immunohistochemistry (IHC) staining. RNA extracted from melanoma tissue was used for RNA sequencing and data analysis.

### RNA Sequencing and Data Analysis

Fresh tumor tissues of melanoma patients were acquired by isolating the sections of metastases from patients without anti-PD-1 therapy. The medical and pathological features of the melanoma patients in this study are shown in **Table 1**. RNA sequencing (RNA-seq) was performed on fresh frozen tumor tissues (n=6). The analysis of RNA-seq data was performed using a two-pass method with STAR. The readings mapped to each gene were enumerated using RSEM, and expression level reading counts were normalized using the FPKM method. Divergent genes were categorized according to standards of *p* < 0.05 and a fold variation > 2.0. The top significantly differentially expressed conveyed genes were separated into the PD-1 high representation and PD-1 low representation groups by the *p* values.

**Table 1:** Clinical and pathological features of puncture melanoma patients in this study.

**Gender**	**Age (at diagnosis)**	**Primary tumor site**	**TNM stage**	**Puncture site**
**Female**	59	Nose	IV	Cervical lymph node
**Female**	55	Left plantar	IV	Chest wall
**Male**	76	Right heel	IV	Lung
**Female**	49	Eye	IV	Liver
**Male**	48	Right heel	III	Inguinal lymph node
**Male**	53	Upper limb	IV	Cervical lymph node

### Lymphocyte Isolation

To obtain single-cell suspension samples for flow cytometry analysis, tissues were isolated from melanoma patients in small pieces. Then, the small pieces were made into tumor suspensions processed using a 70 μm cell filter and centrifuged for 20 min at 2,000 rpm at 4°C using Ficoll-Paque Plus. After centrifugation, the tumor-infiltrating lymphocytes were transferred into a new tube and washed with RPMI-1640 medium including 10% FBS twice. Lysis buffer (BD biosciences, USA) was utilized to remove the red cells when needed. Peripheral blood mononuclear cells (PBMCs) and TILs of mice were quarantined following the same protocols. PBMCs and melanoma TILs were incubated in 1640 medium, and TCR stimulation was performed with anti-CD3, soluble anti-CD28, and IL-2 for three days. To differentiate PD-1 blockade and STAT3 inhibition on Treg cells *in vitro*, we classified Treg cells from PBMCs of healthy donors by flow sorting focusing on CD4^+^CD25^+^CD127^-^ staining cells. Then, Treg cells were cultured in 1640 medium (10% FBS) with 1% penicillin/streptomycin, and later coated the plate with anti-CD3, anti-CD28, and IL-2. IgG, anti-PD-1 (10 ug/mL, Merck, USA), or Stattic (5 uM, MCE, USA) treatments were added as indicated for 48 h.

### Flow Cytometry

Single-cell suspensions were incubated in staining buffer. All antibodies were obtained after BD bioscience, Biolegend, eBioscience, or the R&D system. Surface staining was performed in staining buffer for 30 min at 4°C in the dark. Before stream cytometry, PBMCs and TIL were stimulated with 0.25 mg of brefeldin A for 4 h at 37°C. PBMCs and TILs for extracellular staining were stained with CD4, CD25, CD127, PD-1, and Tim-3. Fixable viability Dye eFluor™ 780 was used for eliminating dead cells. For intracellular staining, cells were first static permeabilized with a record component staining buffer set for 60 min. Besides, single-cell suspensions subjected to intracellular antibody staining for Foxp3, Granzyme B, IL-10, IL-17A, and TGF-β. All samples were incubated in a staining buffer and stored in the dark. Data were collected with a Fortessa or CantoII flow cytometer (BD bioscience, USA) and evaluated applying FlowJo version 10 (FlowJo LLC, USA).

### Mouse Model

All experiments for animals were executed corresponding to the procedures and under the recommendations endorsed from the Institutional Animal Care and Use Committee of Tongji University. C57BL/6 mice aged six weeks were used in the experimental group. B16-F10 cells (5×10^5^ cells) were subcutaneously injected into the limbs of immunocompetent C57BL/6 mice. Tumors were measured using a digital caliper, and their volume was calculated with the formula (width)^2^ ×length/2. The weight of the mice was also taken every two days. After incubation for six days, the mice were subjected to IgG2a as control, anti-PD-1, STAT3 pathway inhibitor Stattic, and a combination of anti-PD-1 with Stattic treatments. The mice were euthanized, and tumors, spleens, and other systemic organs were dissected at the end-point experimental analyses. Tumors and spleens were used to prepare single-cell suspensions for flow cytometry analysis. The main systemic organs were fixed with paraformaldehyde at 4°C for hematoxylin and eosin (H&E) and terminal deoxynucleotidyl transferase-mediated dUTP-biotin nick end labeling assay (TUNEL) discoloration. Mice were randomly assigned into treatment groups.

### Immunohistochemistry Staining

Tissue samples from humans and animals were fixed with paraformaldehyde and embedded with paraffin, then cut into 5 mm sections for further staining. For immunohistochemistry staining, sections were deparaffinized with xylene and rehydrated. Antigen retrieval was performed with a heated antigen unmasking solution (1.0 mM EDTA, 0.05% Tween 20, pH 8.0). Primary antigen Tim-3 (1:1000 for IHC, Cell Signaling Technology, USA) was incubated on the slices overnight at 4°C after 1 h of serum blocking. The DAB chromogen was used for IHC, followed by counterstaining with hematoxylin QS. On the second day, the sections were exposed to the corresponding secondary antibodies for 1 h at room temperature. Slides were digitally scanned using NanoZoomer S210 (Hamamatsu, Hamamatsu, Japan) with a 20X objective, and automated image analysis was performed using NOPview2 software. The comparative expression of Tim-3 was finalized employing ImageJ software within normal (n=3) and melanoma (n=3) tissues.

### Statistical Analysis

Statistical analyses were presented utilizing GraphPad Prism v.8.0 (San Diego, USA). Data points were shown as the mean ± SEM of biological replication. Replication was indicated as * *p* < 0.05 or **p < 0.01 or *** *p* < 0.001 for the total legends. Two-tailed combined or unmatched Student’s t-tests were used for the discovery of significant differences in normally distributed data. For multiple group comparisons, one- or two-way ANOVAs with multiple comparison correction were utilized for defining disagreements. Correlations were determined with Pearson’s coefficient.

## Results

### Expression Levels of HAVCR2 and PDCD1 Are Correlated on Treg Cells in Advanced Melanoma

**Figure 1:**
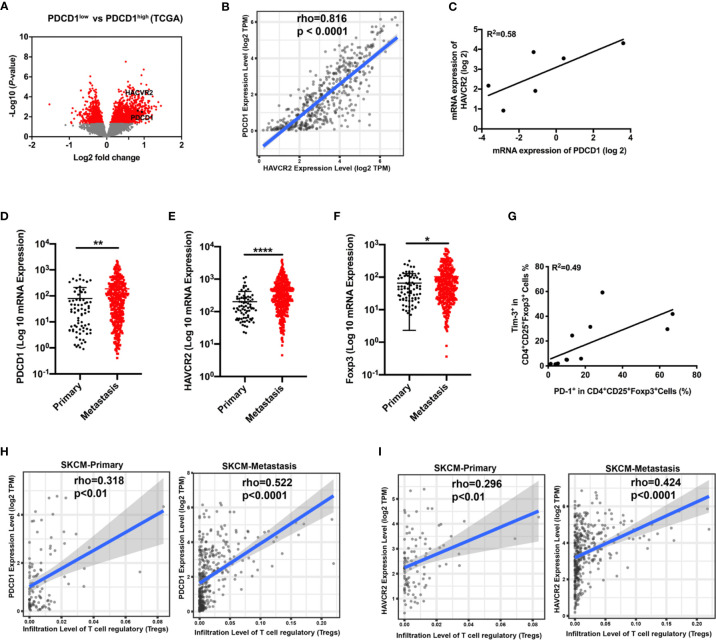
Correlation of PDCD1 expression and HAVCR2 in melanoma. **(A)** Volcano plot showing enrichment of differentially expressed genes in the PD-1^high^ and PD-1^low^ sub-populations from the TCGA dataset (n=472). Significantly different genes (PDCD1 and HAVCR2) are plotted in black (log2 fold change > 0.5, *p* < 0.05). **(B)** Correlation between PDCD1 (PD-1) and HAVCR2 (Tim-3) expression in human melanoma samples from the TCGA dataset (n=472). **(C)** Association between the mRNA level of PDCD1 (PD-1) and HAVCR2 (Tim-3) from Pearson correlation of melanoma biopsies (n=6; R^2^ values by linear regression). **(D–F)** PDCD1 **(D)**, HAVCR2 **(E)**, and Foxp3 **(F)** expression in primary and metastatic melanoma patients from the TCGA dataset. **(G)** Relationship of PD-1 and Tim-3 expression on Treg cells in PBMCs from healthy donors (n=9; R^2^ values by linear regression). **(H, I)** Correlation between PDCD1 **(H)** or HAVCR2 **(I)** expression and Treg cells infiltration in primary and metastasis melanoma patients’ samples from the TCGA datasets. *p ≤ 0.05, **p ≤ 0.01, ****p ≤ 0.0001.

Analysis from the TCGA database demonstrated that the gene HAVCR2 encoding Tim-3 was enriched in differentially expressed genes between the PD-1^low^ and PD-1^high^ cohorts (**Figure 1A**). To evaluate whether HAVCR2 expression was associated with PD-1 expression in melanoma, we performed mRNA expression of PDCD1 encoding PD-1 and HAVCR2 in the tumor biopsies and TCGA databases. Correlation results demonstrated that HAVCR2 showed a positive interaction with PDCD1 in the TME (**Figures 1B, C**). Exploring the clinical significance of PDCD1 and HAVCR2 in melanoma patients, we analyzed the gene expression levels in primary and metastasis melanoma. Expectedly, both PDCD1 and HAVCR2 expression were significantly higher in advanced melanoma compared to primary melanoma in the TCGA datasets (**Figures 1D, E**). We also found that tumor-infiltrated Treg cells were increased in metastasis melanoma (**Figure 1F**). To investigate the dynamic changes of PDCD1 and HAVCR2 on Treg cells in tumor progression, we analyzed the gene expression on tumor-infiltrated Treg cells in TCGA datasets. Interestingly, the checkpoint molecules PDCD1 and HAVCR2 were upregulated in advanced melanoma patients (**Figures 1H, I**). Further corroborating these observations, a positive correlation > 0.4 between Tim-3 and PD-1 expression on human Treg cells was discovered (**Figure 1G**). We analyzed the co-expression of Tim-3 and PD-1 with STAT3 in the melanoma patients and the Treg cells. Results were consistent with the TCGA data. Both Tim-3 and PD-1 showed a positive correlation with STAT3 (**Figure S1**). The clinical characteristics of the related melanoma patients are shown in **Table 1**. Overall, these data suggested that Tim-3 is engaged by PD-1 on Treg cells toward the progression of melanoma.

### Tim-3 Is Upregulated in Treg Cells Upon Anti-PD-1 Treatment

**Figure 2:**
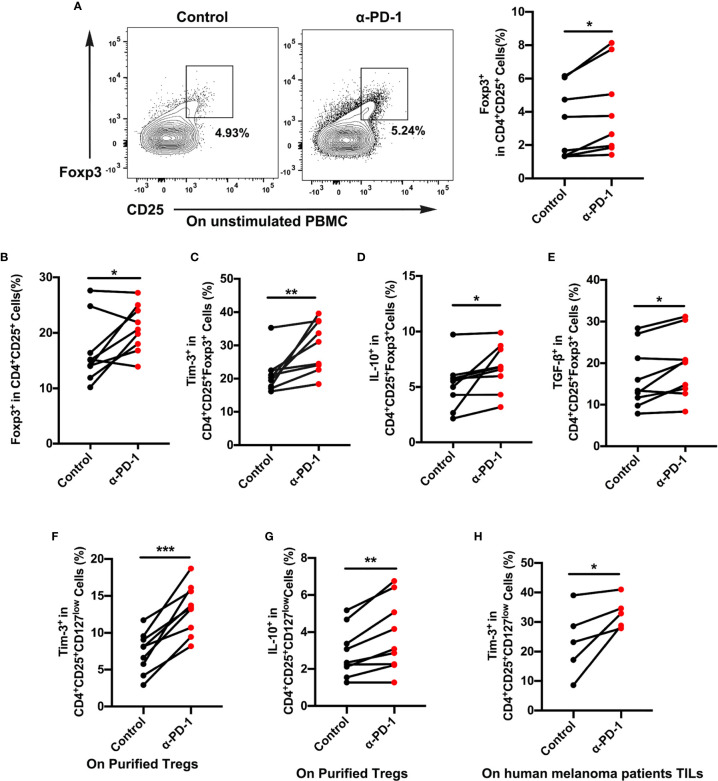
Tim-3 expression upregulated after PD-1 blockade on Treg cells. **(A, B)** Anti-PD-1 antibody (α-PD-1) was given at a concentration of 10 µg mL^-1^ *in vitro* for 48 h. The proportion of Treg cells in unstimulated **(A)** or **(B)** stimulated PBMCs were analyzed (n=9; *p* < 0.05). **(C–E)** Tim-3 expression, IL-10 and TGF-β production were investigated by flow cytometry. Representative Tim-3 expression **(C)**, IL-10 **(D)** and TGF-β secretion **(E)** in Treg cells after stimulation with PD-1 blockade (n=9; *p* < 0.01; *p* < 0.05; *p* < 0.05). **(F, G)** Purified Treg cells were subjected to anti-PD-1 therapy for 48 h. Representative plots representing Tim-3 expression **(F)** and IL-10 **(G)** in Treg cells (n=9; *p* < 0.001). **(H)** Freshly isolated tumor-infiltrating lymphocytes (TILs) from melanoma patients were treated with nivolumab 10 µg mL^-1^ for 48 h. Tumor-infiltrating Treg cells from melanoma patients were analyzed for Tim-3 expression (n=5; *p* < 0.05). Significance was calculated with a *t*-test. All data are presented as the means ± SEM, *p < 0.05; **p < 0.01; ***p < 0.001.

Although PD-1-blocking antibodies have presented excellent effects in melanoma therapy, only a few patients experience ideal therapeutic effects (4, 5). To elucidate the role of immune cells after anti-PD-1 therapy, we assessed the proportion changes of Treg cells in the PBMCs. Results showed a dramatic rise in Treg cell expression, which reflected immune regulation changes after checkpoint blockade therapy (**Figures 2A, B**). To further identify functional differences of Treg cells, we then investigated the expression of Tim-3 on CD4^+^CD25^+^Foxp3^+^ Treg cells from PBMCs or purified Treg cells upon anti-PD-1 antibody. The dynamic expression of Tim-3 on Treg cells was checked to confirm immune suppression after incubation with an anti-PD-1 antibody in the standard medium for 48 h. As shown in **Figure 2C**, a percentage of Tim-3^+^ cells were significantly upregulated in Treg cells after PD-1 blockade (*p* < 0.01). We also found that immune-suppressive cytokines IL-10 and TGF-β were highly increased in Treg cells after anti-PD-1 treatment (**Figures 2D, E**; *p <*0.05). Besides, purified Treg cells from PBMCs were further sorted to completely understand the characteristics of Tim-3 expression on Treg cells. Similarly, flow cytometric data demonstrated that blockade of the PD-1 pathway markedly increased the level of Tim-3 expression on purified Treg cells, suggesting an upregulated immune suppression (**Figure 2F**, *p* < 0.001). Further corroborating these observations, the cytokine IL-10 on purified Treg cells was significantly increased after anti-PD-1 treatment (**Figure 2G**, *p* < 0.01). Moreover, melanoma samples from patients were obtained and analyzed to investigate the levels of Tim-3 and immunosuppressive factors. In melanoma tissues, we found a significant upregulation of Tim-3 on Treg cells after anti-PD-1 therapy *in vitro* (**Figure 2H**, *p* < 0.05), which was similar to PBMCs. Thus, these findings suggest that blocking the PD-1 pathway significantly upregulates Tim-3 expression on Treg cells. Consistent with our previous study, we also found that Tim-3 downregulated after a decrease in the proportion of TGF-β secretion after PD-1 pathway blockade both in PBMCs and melanoma tissues, reflecting the excellent efficacy of anti-PD-1 therapy (**Figures S2A–D**). These results support the hypothesis that Tim-3 expression can be a potential biomarker for predicting tumor-progression and resistance to anti-PD-1 immunotherapy in melanoma patients.

For verification that Treg cells were in a functional state, the proliferation of Treg cells was analyzed after different treatments. It was hoped that Treg cells in the anti-PD-1 group would display a lower proliferation rate than did those in the control group (**Figures S3A, B**), which would suggest a suppressive state of Treg cells after anti-PD-1 therapy. Brought jointly, these results confirmed that Tim-3 expression represented an adaptive response for keeping the suppressive status of Treg cells in response to PD-1 blockade.

**Figure 3:**
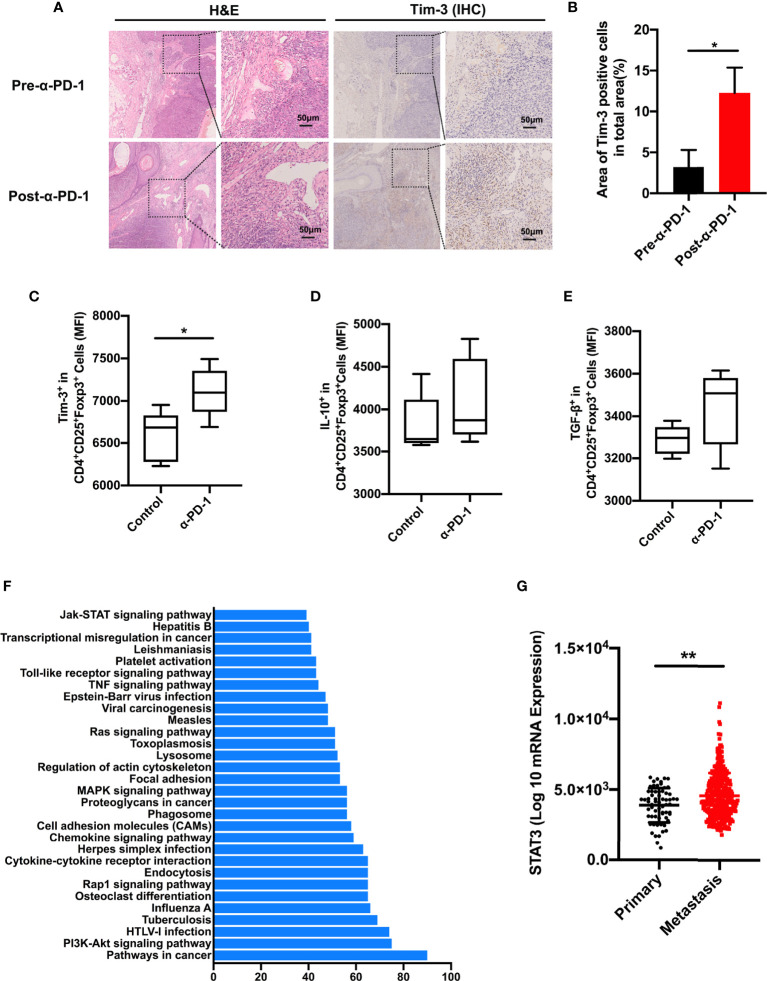
STAT3 pathway involved in PD-1 expression. **(A, B)** H&E and IHC for Tim-3 expression levels in anti-PD-1 resistance melanoma patients’ tissue samples (n=3). Representative images from three patients displayed positive Tim-3 staining in stromal regions **(A)**. Scale bar = 50 μm. Quantification of positive cells in tumor sections using ImageJ software **(B)**. Significance was calculated with a *t*-test, and all data are presented as the means ± SEM. **(C, E)** Tim-3 expression **(C)**, IL-10 **(D)**, and TGF-β **(E)** production in Treg cells were investigated by flow cytometry after anti-PD-1 treatment in the B16-F10 melanoma mouse model. **(F)** Significantly enriched pathways terms of differentially expressed genes in the PD-1^high^ and PD-1^low^ sub-populations in melanoma. The top 30 functional and signaling pathway enrichment were conducted using the online website DAVID. **(G)** STAT3 expression in primary and metastatic melanoma patients from the TCGA dataset. *p ≤ 0.05 and **p ≤ 0.01.

To confirm that the upregulation of Tim-3 occurred after anti-PD-1 immunotherapy, we analyzed the immunohistochemistry (IHC) staining using samples from anti-PD-1 nonresponding melanoma patients. As expected, the checkpoint molecule Tim-3 was increased upon PD-1 immunotherapy in advanced melanoma patients (**Figures 3A, B**). These results were corroborated with the aforementioned data. To identify the role of Tim-3 expression on tumor growth, we established an immunocompetent skin melanoma model using B16-F10 cells. Our data identified that the PD-1 pathway blockade upregulated the proportion of Tim-3 expression on melanoma-associated Treg cells (**Figure 3C**). Moreover, we observed a slight rise in the secretion of IL-10 and TGF-β with anti-PD-1 therapy *in vivo* compared to control melanoma mice, implying an enhanced immune suppression (**Figures 3D, E**). These results were similar to the previous *in vitro* data. Taken together, these results confirmed that Tim-3 expression represents an adaptive response to maintain the suppressive status of Treg cells in response to PD-1 blockade.

To investigate the molecular mechanism of Tim-3 upregulation upon anti-PD-1 treatment, we evaluated the candidate differentially expressed genes between the PD-1^low^ and PD-1^high^ cohorts using the online website DAVID. As shown in **Figure 3F**, the top 30 related functional and signaling pathways were identified. These genes were enriched in pathways related to cancers, PI3K-Akt signaling, cytokine-cytokine receptor interaction, Jak-STAT signaling pathway, and so on (**Figure 3F**). The STAT3 pathway was found to present an essential role in the inflammatory response of adaptive immune cells. To determine the clinical significance of the STAT3 gene, we performed STAT3 expression in TCGA melanoma datasets (**Figure 3G**). Its significance was not hard to confirm as STAT3 expression is higher in metastasis sites than in primary tissue (**Figure 3G**). Above all, these data proposed that STAT3 could mediate tumor immunity upon PD-1 blockade.

### Downregulation of STAT3 Decreases Tim-3 Expression in Treg Cells

**Figure 4:**
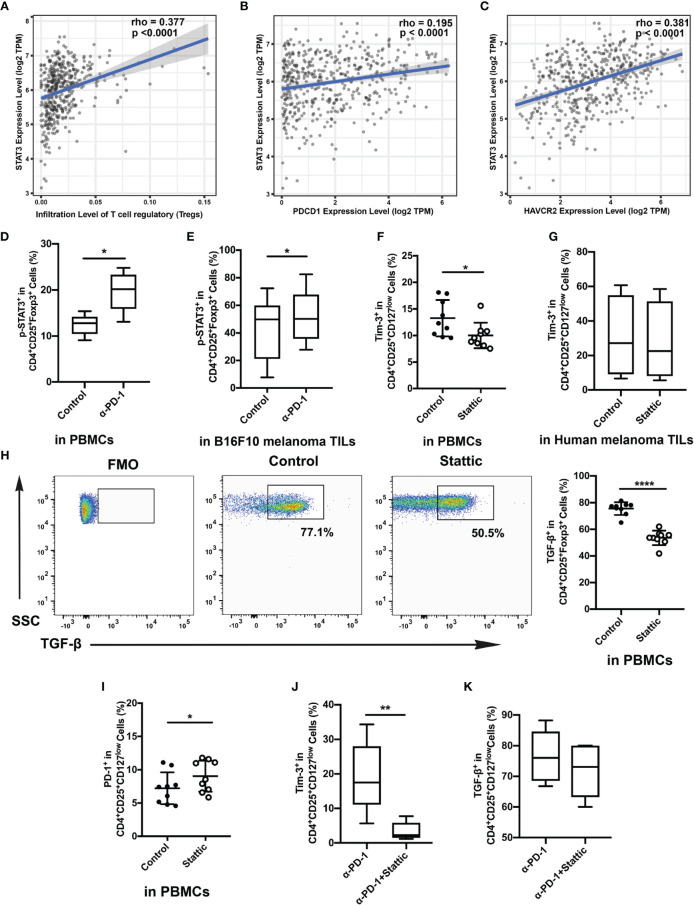
Relationship of STAT3 and Tim-3 in melanoma. **(A–C)** Correlation between STAT3 expression and tumor-infiltrated Treg cells **(A)**, the expressed genes PDCD1 **(B)** and HAVCR2 **(C)** in human melanoma samples from the TCGA dataset. **(D, E)** p-STAT3 expression after anti-PD-1 treatment both *in vitro* **(D)** and *in vivo* **(E)**. **(F, G)** Proportion of circulating Treg cells treated with STAT3 inhibitor compared to that of the control group. Analysis of the phenotypic expression of Tim-3 on Treg cells in PBMCs **(F)** and human melanoma TILs **(G)**. **(H, I)** PD-1 expression **(H)** and TGF-β **(I)** production on Treg cells after the STAT3 pathway blockade were also analyzed. Significance was calculated with a *t*-test; all data are presented as the means ± SEM, *p < 0.05 and **p < 0.01. **(J, K)** Analysis of the expression of Tim-3 **(J)** and production of TGF-β **(K)** on purified Treg cells after anti-PD-1 treatment with Stattic. Significance was calculated with a *t*-test; all data are presented as the means ± SEM, *p < 0.05, **p < 0.01, and ****p ≤ 0.0001.

The STAT3 pathway performs the core part of the inflammatory response of adaptive immune cells. And STAT3 also acts both as a co-transcription pathway for Foxp3 and a mediator for IL-10 in Treg cells (17). To further address the molecular mechanisms triggered in Treg cells upon Tim-3 upregulation, we evaluated a crucial mediator of carcinogenesis STAT3 through immunosuppression in melanoma. To identify the relationship between tumor-infiltrated levels of Treg cells and STAT3, the melanoma cohort of the TCGA dataset was filtered and analyzed by Spearman correlation analysis. As shown in **Figure 4A**, STAT3 showed a high correlation with Treg cells in melanoma datasets. Moreover, PDCD1 and HAVCR2 exhibited a positive correlation with STAT3, but the association in PDCD1 was weak (r_pearson_= 0.195; 0.381, respectively) (**Figures 4B, C**). These findings support that STAT3 could act as a crucial mediator of Treg cell-associated checkpoint receptor expression in the TME. Besides, we also found that the expression of the level of p-STAT3 was increased in the anti-PD-1 group with increased Tim-3 expression both *in vitro* and *in vivo* (**Figures 4D, E**). According to the above results, we hypothesized that Tim-3 was upregulated through the STAT3 pathway in Treg cells after the PD-1 blockade.

A small-molecule inhibitor of p-STAT3 (Stattic) was used to confirm whether Tim-3 was regulated after anti-PD-1 therapy. Cells were incubated with the inhibitor for 48 h after anti-CD3/28 stimulation. Flow analysis indicated that Stattic reduced the level of Tim-3 compared with the vehicle controls after TCR stimulation (**Figure 4F**). Moreover, we also performed a flow cytometric analysis on Tim-3 on Treg cells upon depletion of STAT3 in melanoma tissues (**Figure 4G**). Similar to the results, we also found a slight decrease in Tim-3 expression in melanoma-infiltrated Treg cells (**Figure 4G**), suggesting that inhibited STAT3 could abrogate Tim-3 expression in Treg cells. However, we also found that PD-1 expression upregulated upon exposure to Stattic (**Figure 4H**). To determine the impact of STAT3 downregulation on Treg cells function, we used Treg cells to analyze the production of TGF-β by stimulating them with brefeldin A *in vitro* (**Figure 4I**). The percentage of TGF-β in Treg cells was found to decrease after STAT3 depletion (**Figure 4I**, *p <*0.0001). Melanoma-infiltrated Treg cells exhibited a similar *in vitro* Tim-3 increasing trend in STAT3 pathways blockade. These results strongly suggested that STAT-3 downregulation dampened the level of Tim-3 expression in Treg cells, leading to decreased immunosuppression. Furthermore, to confirm the therapeutic efficacy of combining STAT3 downregulation with anti-PD-1, we treated Treg cells with Stattic after anti-PD-1 treatment for 48 h *in vitro* (**Figures 4J, K**). To our expectations, STAT3 downregulation after anti-PD-1 therapy decreased the expression of Tim-3 in Treg cells (**Figure 4J**). The immunosuppressive cytokines of TGF-β were also analyzed in Treg cells (**Figure 4K**). A slight decrease of TGF-β production was found in Treg cells with PD-1 and STAT3 pathway blockades (**Figure 4K**). Altogether, these findings confirmed that targeting STAT3 could abrogate the immunosuppression of Treg cells, providing a potential target in melanoma treatment.

### STAT3 Inhibitor Enhances the Efficacy of Anti-PD-1 in Melanoma

**Figure 5:**
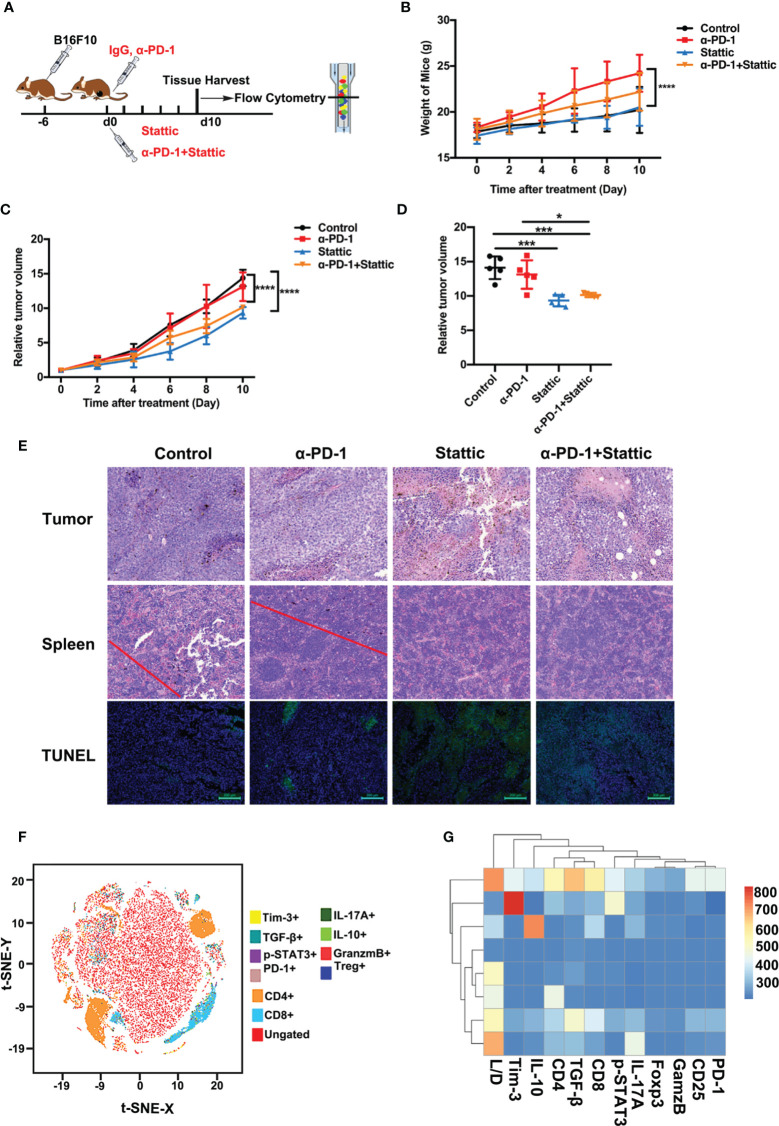
STAT3 inhibitor enhanced the efficacy of anti-PD-1 in a melanoma model. **(A)** Schematic illustration of different treatments in the melanoma mouse model. **(B)** Weight curve of B16-F10 tumors from mice administered with different treatments (n=5*; p <*0.0001). **(C)** Relative tumor volume curve of B16-F10 tumors after treatment with anti-PD-1 alone, Stattic alone, anti-PD-1 plus Stattic, and IgG isotype control (n=5; *p <*0.0001). **(D)** Relative tumor volume at terminal time of experiments with four different treatments (n=5; *p <*0.05; *p <*0.001; *p <*0.001). **(E)** Tumor necrosis in tumor sections as indicated by hematoxylin and eosin (H&E) staining. Tumor metastasis to the spleen was assessed by H&E staining. Tumor apoptosis in tumor sections was examined using TUNEL staining. Scale bar = 50 μm or 100 μm. **(F)** tSNE plots of CD8^+^ T cells of spleen tissues from B16-F10 tumor-bearing mice. **(G)** Heat map of the tumor-associated gene signature mapped to the different tumor treatment, representing the spleen tissues and CD8^+^ T cells subsets from B16-F10 tumor-bearing mice. *p ≤ 0.05, ***p ≤ 0.001,****p ≤ 0.0001.

For the identification of Tim-3 expression in tumor expansion, we established immunocompetence for the skin melanoma model using B16-F10 cells. The generated melanoma mouse models were obtained after four different treatments: IgG for control, a PD-1 antibody, Stattic, and anti-PD-1 combination with Stattic (**Figure 5A**). First, we found that mouse bodyweight in the anti-PD-1 treatment group increased during tumor progression, while STAT3 downregulation did not alter the weight (**Figure 5B**). Expectedly, in the PD-1 blockade group, anti-PD-1 treatment did not alter tumor growth in the melanoma model (**Figure 5C**). Interestingly, in the combination group, we also found a marked reduction in tumor growth in the B16-F10 melanoma mouse model (**Figure 5D**, *p* < 0.0001). Under these conditions, STAT3 downregulation significantly increased the anti-PD-1 efficacy in melanoma. *In vivo* depletion experiments also demonstrated that the STAT3 pathway blockade was essential in inhibiting tumor progression, suggesting a potential target therapy.

To confirm the efficacy of combining STAT3 blockade with anti-PD-1 in the C57BL/6 animal model, we performed H&E and TUNEL staining of tumors. Tumor metastasis and apoptosis were evaluated by H&E and TUNEL staining experiments. A significant increase for tumor cell necrosis in melanoma was observed in the STAT3 inhibitor group with anti-PD-1 therapy, indicating excellent therapy efficacy (**Figure 5E**). Moreover, H&E staining showed no evident metastasis of spleens in the Stattic and anti-PD-1 co-treatment group (**Figure 5E**). TUNEL staining results confirmed that the frequency of apoptosis cells increased after STAT3 depletion with blockade (**Figure 5E**). Therefore, these data suggest that Stattic with PD-1 blockade inhibits tumor growth and metastasis, providing potential therapy for melanoma treatment.

To determine the impact of STAT3 downregulation on anti-melanoma immune response, we analyzed the cell phenotype and cytokine expression pattern in spleen tissue using flow cytometry (**Figure 5F**). We found a gradient of low to elevated degrees of immune cells in the spleen (**Figure 5F**). As expected, the flow cytometric study elaborated that CD4^+^ and CD8^+^ T cells were exceedingly concentrated within the cell clusters (**Figure 5F**). Next, we explored CD8^+^ T cell-driven cytokines and multi-inhibitory receptors for disclosing the fundamental downstream mechanisms. Analysis of CD8 subsets, assessed by the manifestation of PD-1, Tim-3, IL-10, TGF-β, granzyme B, and the levels of p-STAT3, indicated that Tim-3 and TGF-β were differentially expressed in these subsets with a distinct transcriptional signature compared to that of other subsets (**Figure 5G**). These results suggest a potential mechanism of Tim-3 regulation in anti-melanoma immunity.

### Blockade STAT3 Pathway Promotes Anti-Melanoma Immune Response

**Figure 6:**
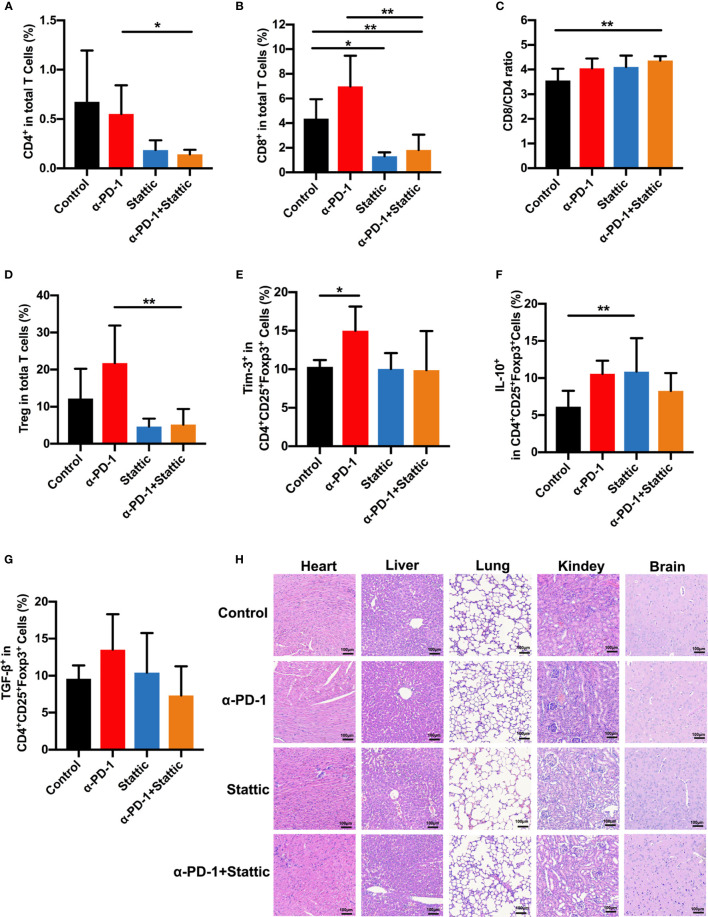
STAT3 downregulation promotes antitumor immune response. **(A–C)** Analysis of intratumoral CD4^+^ and CD8^+^ T cells in melanoma models. The proportion of intratumoral CD4 **(A)** cells and CD8 **(B)** T cells and CD8^+^/CD4^+^ T cells ratio **(C)** in response to anti-PD-1 in comparison to anti-PD-1 and Stattic, Stattic, and control groups. **(D)** Percentages of CD4^+^CD25^+^Foxp3^+^ Treg cells among total cells in melanoma (n=5). **(E–G)** percentage of positive Tim-3 expression **(E)**, IL-10 **(F)** and TGF-β **(G)** production in Treg cells after different treatment in a mouse model. **(H)** Systematic toxicity of anti-PD-1 and Stattic in melanoma models. Organ toxicity was assessed by H&E staining. Scale bar = 100 μm. *p ≤ 0.05 and **p ≤ 0.01.

To examine the influence of STAT3 downregulation upon the anti-tumor immune response, we analyzed tumor-infiltrated lymphocytes that were studied in melanoma mice. Interestingly, STAT3 depletion and anti-PD-1 therapy decreased the percentages of CD8^+^ and CD4^+^T cells, leading to an increased CD8/CD4 ratio in STAT3 downregulation and PD-1 blockade combinational treatment in melanoma according to monitoring mice in the PD-1 blockade group (**Figures 6A–C**). Similar results were observed in STAT3 downregulation when compared to the control group (**Figures 6B, C**). Moreover, *in vivo* experiments confirmed a marked reduction in the percentages of Treg cells after treatment with a STAT3 inhibitor with an anti-PD-1 antibody (**Figure 6D**). The findings implied that Treg cells were essential in the inhibition of tumor growth (**Figure 6D**, *p* < 0.05). To understand how the STAT3 pathway regulates the tumor infiltrated Treg cells, we evaluated the expression of Tim-3 and immunosuppressive cytokines. Our data identified that the PD-1 pathway blockade upregulated the proportion of Tim-3 expression on melanoma-associated Treg cells (**Figure 6E**). While combining STAT3 downregulation and anti-PD-1 decreased the manifestation of Tim-3 on Treg cells during the anti-tumor response (**Figure 6E**). Moreover, we observed a slight rise in the secretion of TGF-β and IL-10 in the anti-PD-1 group compared to the IgG2a group (**Figures 6F, G**), which was similar to *in vitro* data. Furthermore, flow cytometric analysis demonstrated that STAT3 inhibitor with PD-1 treatment weakened the secretion of TGF-β and IL-10 on Treg cells compared to anti-PD-1-treated melanoma mice, implying an enhanced anti-tumor immunity (**Figures 6F, G**). These data suggested that combination therapy with anti-PD-1 and a STAT3 pathway inhibitor could promote anti-tumor immunity and suppress tumor progression. Thus, the above data suggested that Tim-3 expression on Treg cells in the TME was STAT3-dependent, providing further support for STAT3 as a target and enhancing the immunotherapy for patients suffering from melanoma.

To confirm the role of combination STAT3 downregulation and anti-PD-1 treatment on CD8^+^ T cells, we analyzed the cell phenotype and cytokine expression pattern of CD8^+^ T cells in melanoma (**Figures S4A–C**). Interestingly, there was a reverse trend in CD8^+^ T cells compared with Treg cells after different treatments (**Figures S4A–C**). STAT3 downregulation with and without anti-PD-1 therapy was related to an increased expression of Tim-3 in CD8^+^ T cells as an activation towards CD8^+^T cells (**Figure S4A**). Moreover, the STAT3 blockade induced secretion of IL-10 and TGF-*β* in CD8^+^ T cells compared that in IgG-treated mice (**Figures S4B, C**). Our finding indicated that STAT3 presents a reverse event in the cross-talk between Treg cells and CD8^+^ T cells. Altogether, these results strongly confirmed that STAT3 downregulation dampened Treg function in melanoma, enhanced the anti-tumor immunity. Based on the above data, our work demonstrates that Tim-3 was upregulated through the STAT3 pathway in checkpoint receptors inhibitor therapy.

Furthermore, to investigate the systemic toxicity after different treatments, we conducted a H&E staining experiment on the main organs, including the heart, liver, lungs, kidneys, and brain. There was no apparent toxicity in the main organs after the end of treatment (**Figure 6H**). Based on these data, our work suggests a safe therapy by combining using p-STAT3 inhibitor Stattic and anti-PD-1 to regulate tumor immune therapy.

## Discussion

Our study provides evidence that the STAT3 pathway behaves as an immune mediator, increasing Tim-3 expression in the TME and affecting the response to anti-PD-1 treatment in melanoma. Recently, Tim-3 has been identified as being present in the core part of effector T cells and cytokine fabrication within PBMCs and TILs (23, 24). In human patients with melanoma, high Tim-3 expression was linked to the exhaustion of tumor-infiltrating CD8^+^ T cells and Tim-3 monoclonal antibodies reverse tumor-induced T cells (25, 26). However, Tim-3 is not usually present in Treg cells, except in those in a TME upon stimulation of tumor-associated antigens (27). A study showed that almost 60% of tumor-infiltrating Treg cells in diseased persons with lung cancer expressed Tim-3, promoting tumor suppression (28). Tim-3^+^ Treg cells were found to present much more tumor immunosuppression than Tim-3^-^ Treg cells in lung cancer patients, with the secretion of IL-10 and granzymes (29). Compared with Tim-3^-^ Treg cells, Tim-3^+^ Treg cells presented a high suppressive capacity by inhibiting the proliferation of conventional T cells (30). Tim-3^+^ has been demonstrated as a marker of Treg cells in immunosuppression, dampening the effector function of CD8^+^ T cells (31). Tim-3^+^ TIL Treg cells were much more suppressive than PD-1 higher expression Treg cells, which were known as exhausted Treg cells (32). Moreover, Tim-3 overexpression has been identified in various diseases, including head and neck cancer and bladder cancer (33–35). High Tim-3 expression was related to the poor survival of patients with many types of tumors in a clinical study (36). However, its role in Treg cells on melanoma has not been described. Interestingly, the obtained results suggested that a low response to PD-1/PD-L1 in lung cancer patients was associated with higher Tim-3 expression (37). The previous study identified that effective PD-1 blockade decreased Tim-3 expression in Treg cells in HNSCC tumors (14). While the dynamic changes of Tim-3 in Treg cells in anti-PD-1 nonresponse melanoma patients have not been elucidated. Herein, we demonstrated that the upregulation of Tim-3 on Treg cells correlated with anti-PD-1 resistance melanoma patients. Notably, the proportion of Tim-3 expression in Treg cells was upregulated after PD-1 blockade in a murine melanoma model. *In vitro*, we also observed an increased production of suppressive factors (TGF-β) after anti-PD-1 therapy, which was related to the upregulation of Tim-3 expression in Treg cells. These results highlighted the Tim-3 expression in Treg cell-induced tumor immunosuppression in the TME. Our study also provides a potent combination of immune checkpoint receptors to enhance the therapeutic response using PD-1 and Tim-3 antibodies in cancer.

STAT3 is a member of the signal transduction and activator of transcription family mediating tumor proliferation, progression, metastasis, and immunity in the tumor microenvironment (38). The JAK-STAT signaling pathway regulates the transcription of DNA and expression of genes involved in cancer behavior: tumor apoptosis and tumor angiogenesis (39, 40). Evidence has demonstrated that the JAK/STAT3 pathway mediates tumor angiogenesis by increasing expression and activation of environmental hypoxia in the TME (41). Therefore, inhibiting STAT3 pathways can significantly reduce vascular endothelial growth factors, which could inhibit tumor growth. Our work indicated that the downregulation of STAT3 decreased melanoma growth in the murine mouse model. It is hard to deny the potential impact of STAT3 downregulation on tumor angiogenesis inhibition.

Herein, results have demonstrated that STAT3 downregulation in melanoma markedly reduced the proportion of tumor-infiltrated Treg cells. Although the role of Treg cells in immunosuppression has been well illustrated, the activities of Treg cells under the PD-1 blockade are still unknown. Indeed, this study demonstrated the role of STAT3 in the modulation of tumor-infiltrated Treg cells *in vitro* and *in vivo* melanoma mouse models. The STAT3 pathway has been found to act as a co-transcription pathway for Foxp3 and to mediate other associated STAT3 targets, including IL-10, in Treg cells (17). Previous studies have shown that STAT3 expression in Treg cells is associated with a suppressive function (19). To explore the crucial role of Treg cells, we conducted a flow analysis of TILs composition. Treg cells in the B16-F10 melanoma tumor model showed increased infiltration after anti-PD-1 while in a low CD8/CD4 ratio, suggesting immune escape in the checkpoint blockade. Besides, our results demonstrated that p-STAT3 in Treg cells upregulated after increased TGF-β production in animal models of melanoma after the PD-1 pathway blockade. Thus, these data confirmed that STAT3 expression is upregulated to adaptive resistance to PD-1 blockade in Treg cells in the TME. These data highlighted the essential role of STAT3 in the regulation of melanoma immune escape. Thereby, blockading the STAT3 pathway can be an excellent target to promote immunotherapy response. Treg cells can induce immunosuppression by secreting the molecules TGF-β *via* contact-dependent or contact-independent mechanisms (14, 42). TGF-β is synthesized and secreted within the cells and promotes Treg cell development (43, 44). To understand the molecular mechanisms of Treg upon anti-PD-1 therapy, we analyzed the production of immunosuppressive factors on Treg cells, such as TGF-β. We observed that purified Treg cells exhibited the increased production of TGF-β after PD-1 blockade *in vitro*; these molecules play an essential role in tumor immune escape. Moreover, TGF-β was found to be markedly reduced by STAT3 depletion, resulting in a decrease of Treg cells. Further studies on the role and mechanism of TGF-β on Treg cells regulated by the STAT3 pathway need to be explored in the future.

However, the molecular mechanism through which Tim-3 levels in Treg cells are mediated by anti-PD-1 therapy remains unclear. Literature has demonstrated that Tim-3 expression is associated with STAT3 expression and phosphorylation in immunosuppression (45). Herein, this work provides evidence that the upregulation of Tim-3 played a crucial role in anti-PD-1 resistance melanoma patients. To explore whether the immune checkpoint expression of Tim-3 changes through STAT3 pathways, we analyzed the expression and correlations of these genes. The examination of the TCGA data, IHC results, and melanoma gene profiles in our study demonstrated a strong relationship among Tim-3 (HAVCR2), PD1 (PDCD1), and STAT3 expression. These findings identified the vital role of STAT3 in the expression of Tim-3. Thus, inhibiting STAT3 may demonstrate an excellent activity on the downregulation of Tim-3, increasing the immune activities in melanoma. We further assessed the dynamic expression of Tim-3 on Treg cells to confirm the regulation of STAT3 after Stattic therapy. Expression of Tim-3 decreased with PD-1 upregulation on Treg cells after the STAT3 pathway blockade *in vitro*. These data suggested that the STAT3 pathway mediated the expression of Tim-3 on Treg cells in melanoma. According to the above results, we hypothesized that Tim-3 was upregulated through the STAT3 pathway after the PD-1 blockade to decrease the anti-PD-1 response. In melanoma models, the expression of Tim-3 on Treg cells decreased, but there was no significant change in Stattic-treated mice compared with the control mice. These data confirmed that Tim-3 was mediated by stimulating TCR signaling to STAT3 pathways during PD-1 blockade. But it remains to be explored in the future whether STAT3 inhibitors act directly on Treg cells, which subsequently modulate the TME through tumor-associated antigens. In our study, there was no observed synergistic effect between STAT3 inhibitor Stattic and anti-PD-1. Former research showed that the effect of combination treatment containing anti-PD-1 therapy on melanoma was not significant in the short term when compared to anti-PD-1 alone after tumor formation for 14 days (46). Another study focused on the antitumor activity of combinations of cytotoxic chemotherapy and immune checkpoint inhibitors of various mouse models. Results showed that different strains of mice react differently at a systemic level to the therapeutic agents used for a limited time (47). As presented here, the combination of a STAT3 inhibitor and anti-PD-1 identified a significant benefit for synthesis therapy. The results from the C57BL/6 tumor samples showed that STAT3 targeting combined with PD-1 blockade leads to a high CD8/CD4 ratio and a decreased percentage of Treg cells. Although the combination therapy presented excellent antitumor immunity, the efficacy of other cancer models has not been identified. The information reinforced that Tim-3 was mediated by stimulating TCR signaling to STAT3 pathways during PD-1 blockade in melanoma, providing further support for combination STAT3 inhibitor and anti-PD-1 therapy for patients suffering from melanoma. But it remains to be explored in the future whether STAT3 inhibitors act directly on Treg cells, which consequently alter the TME through tumor-associated antigens.

## Conclusions

Finally, our current work addressed that Tim-3 was the key character within Treg cells in anti-PD-1 resistance of melanoma. In this study, we revealed a potential mechanism by the STAT3 pathway, which regulated Tim-3 manifestation upon Treg cells in the immune microenvironment. Targeting Treg cells with the STAT3 inhibitor Stattic decreases Tim-3 articulation mutually *in vitro* and *in vivo*, increasing the immunotherapy efficacy of anti-PD-1. Further observations and experiments should be performed to address the mechanism of patient responses to combination therapies in melanoma. Moreover, the potential immunotherapy effect of combination STAT3 antagonists with other checkpoint receptor inhibitors and other immune cells like γδTreg cells and M2 macrophages involved in synthetic therapy need to be explored. In summary, our work supports that Tim-3 expression modulated the immune escape in melanoma as a biomarker for predicting the immunotherapy response. Also, we identified that the transient small-molecule reticence of the STAT3 pathway may dramatically expand the usefulness of anti-PD-1 within melanoma. Therefore, our work provides a potential therapy for STAT3 as a target in melanoma treatment, supporting combining anti-PD-1 and STAT3 antagonists as excellent therapy.

## Data Availability Statement

The original contributions presented in the study are included in the article/**Supplementary Material**. Further inquiries can be directed to the corresponding authors.

## Ethics Statement

The studies involving human participants were reviewed and approved by the Ethics Committee of Shanghai Tenth People’s Hospital (2019K39). The patients/participants provided their written informed consent to participate in this study. The animal study was reviewed and approved by institutional Animal Care and Use Committee of Tongji University.

## Author Contributions

LH performed and analyzed the animal examination and was a major contributor in writing the manuscript. YX analyzed and interpreted the patient data regarding the immunity of melanoma. JF performed the histological examination of the tumor samples and other organs. WL acquired and analyzed the flow data and reviewed the manuscript. ZL and LH developed the methodology of this study. JC, ZL, and QX conducted the conception and design of the manuscript. All authors contributed to the article and approved the submitted version.

## Funding

This work was supported by grants from the Shanghai Sailing Program (No: 19YF1438300), the Pujiang Fostering Program of Shanghai Tenth Peoples’ Hospital (No: 040118024), the National Natural Science Fostering Foundation of Shanghai Tenth Peoples’ Hospital (No: DS040317061), the Industry-university-research-medicine Project of Shanghai Science and Technology Commission (No: 18DZ1910102), and the National Natural Science Foundation of China (No: 81702311, 81803090, and 81902896).

## Conflict of Interest

The authors declare that the research was conducted in the absence of any commercial or financial relationships that could be construed as a potential conflict of interest.

## Supplementary Material

Supplementary Figure 1 Co-expression of Tim-3 and PD-1 with STAT3 in the melanoma patients and Treg cells. **(A, B)** Co-expression of PDCD1 (PD-1) and HAVCR2 (Tim-3) with STAT3 in human melanoma samples from the TCGA dataset (n=472). **(C, D)** Relationship of PD-1/Tim-3 expression and STAT3 on melanoma patients TIL cells (n=6; R^2^ values by linear regression). **(E, F)** Relationship of PD-1/Tim-3 expression with STAT3 on Treg cells in PBMCs from healthy donors (n=12; R^2^ values by linear regression).

Supplementary Figure 2 Tim-3 expression downregulated after PD-1 blockade on Treg cells. **(A–C)** An anti-PD-1 antibody (α-PD-1) was given at a concentration of 10 µg mL^-1^ *in vitro* for 48 h. The proportion of Tim-3 expression **(A)**, IL-10 **(B)** and TGF-β **(C)** production in Treg cells were analyzed (n=9; *p* < 0.05; *p* < 0.001). **(D)** TILs-infiltrated Treg cells from melanoma patients were analyzed for Tim-3 expression (n=7; *p* < 0.01).

Supplementary Figure 3 The proliferation of Treg cells after different treatments. **(A)** Representative histogram of CFSE-labeled human peripheral Treg cells after different treatment (unstimulated, stimulated, and anti-PD-1). **(B)** Proportion of proliferation cells are shown (n=4; *p* < 0.05; *p* < 0.0001). Significance was calculated with one-way ANOVA, and all data are presented as mean ± SEM.

Supplementary Figure 4 Analysis of intratumoral CD8^+^ T cells and the associated phenotype and chemokine profile *in vivo*. (**A-C)** MFI of Tim-3 **(A)**, IL-10 **(B)**, and TGF-β **(C)** determined in CD8^+^ T cells was assessed by flow cytometry. Two-way analysis was performed to assess statistical significance between different groups (n=5; *p* < 0.05; *p*< 0.01).

## Abbreviations

PD-1, Programmed cell death-1; TME, Tumor microenvironment; Tim-3, T cell immunoglobulin and mucin-domain containing-3; Treg, Regulatory T; HNSCC, Head and neck squamous cell, TILs, Tumor-infiltrating lymphocytes; STAT3, Signal transduction and activator of transcription 3; Foxp3, Forkhead box P3; IL-10, Interleukin-10; TGF-β, Transforming growth factor; ATCC, American Type Culture Collection; DMEM, Dulbecco’s Modified Eagle Medium; FBS, Fetal Bovine Serum; IHC, Immunohistochemistry; PBMCs, Peripheral blood mononuclear cells; H&E, Hematoxylin and Eosin; TUNEL, dUTP-biotin nick end labeling assay; TCGA, The Cancer Genome Atlas; RNA-seq, RNA sequencing.
