# Supplementary figures and images for: Targeting STAT3 Abrogates Tim-3 Upregulation of Adaptive Resistance to PD-1 Blockade on Regulatory T Cells of Melanoma
Source: Front Immunol. 2021 Apr 15;12:654749. doi: 10.3389/fimmu.2021.654749 (PMC8082190; doi:10.3389/fimmu.2021.654749)

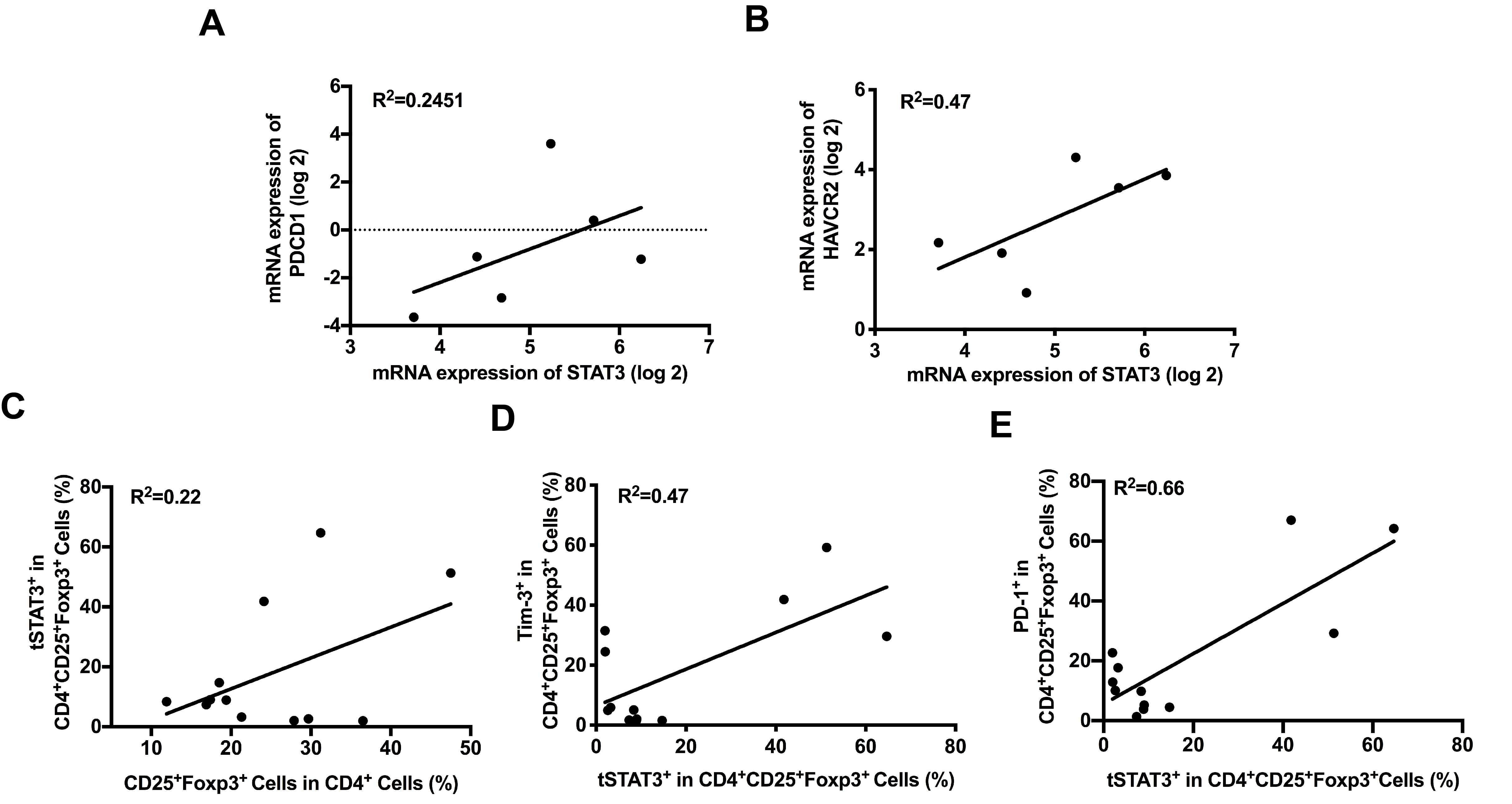

Supplement: Supplementary Figure 1 — Co-expression of Tim-3 and PD-1 with STAT3 in the melanoma patients and Treg cells. (A, B) Co-expression of PDCD1 (PD-1) and HAVCR2 (Tim-3) with STAT3 in human melanoma samples from the TCGA dataset (n=472). (C, D) Relationship of PD-1/Tim-3 expression and STAT3 on melanoma patients TIL cells (n=6; R2 values by linear regression). (E, F) Relationship of PD-1/Tim-3 expression with STAT3 on Treg cells in PBMCs from healthy donors (n=12; R2 values by linear regression). [file Image_1.jpg]

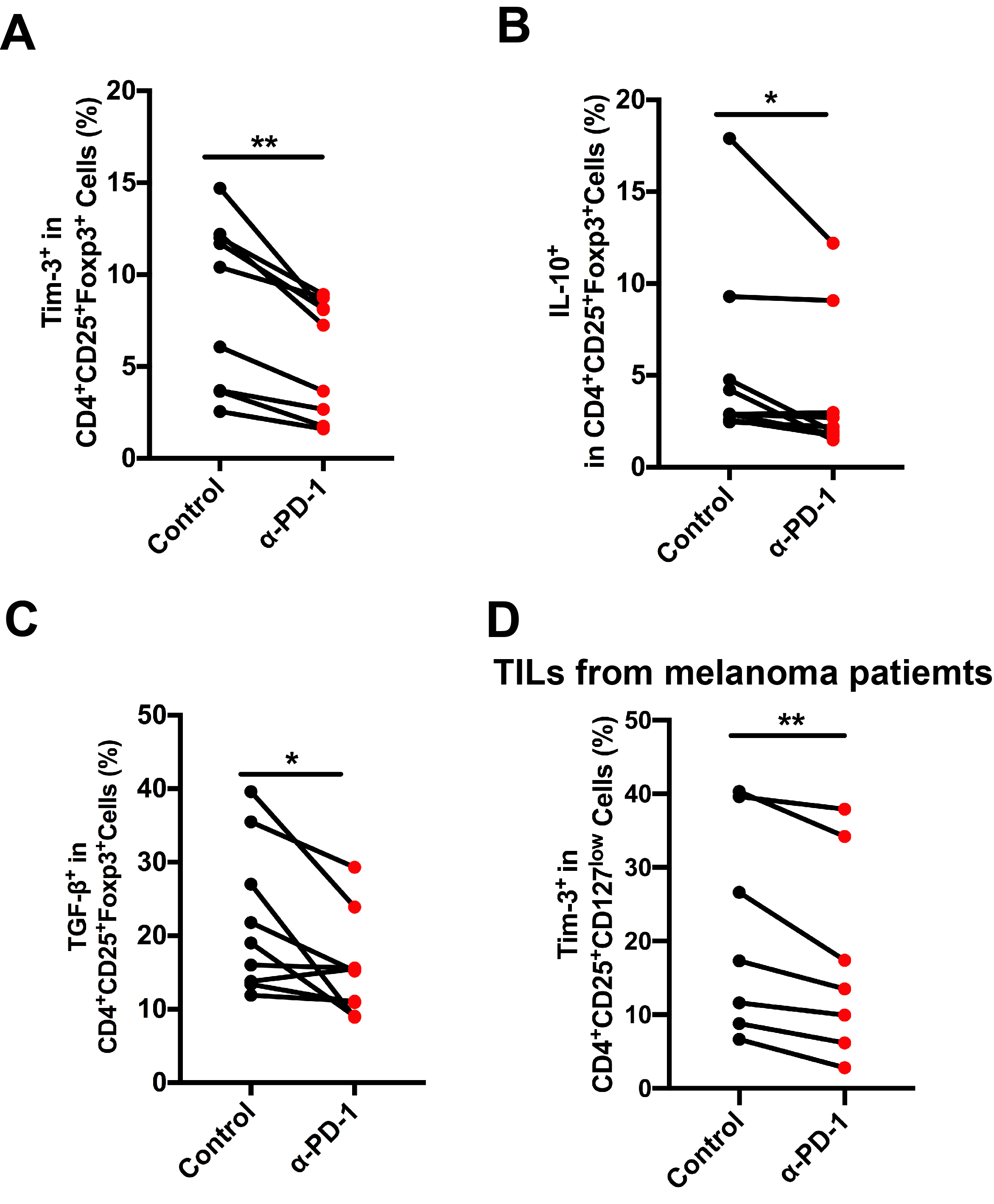

Supplement: Supplementary Figure 2 — Tim-3 expression downregulated after PD-1 blockade on Treg cells. (A–C) An anti-PD-1 antibody (α-PD-1) was given at a concentration of 10 µg mL-1 in vitro for 48 h. The proportion of Tim-3 expression (A), IL-10 (B) and TGF-β (C) production in Treg cells were analyzed (n=9; p < 0.05; p < 0.001). (D) TILs-infiltrated Treg cells from melanoma patients were analyzed for Tim-3 expression (n=7; p < 0.01). [file Image_2.jpg]

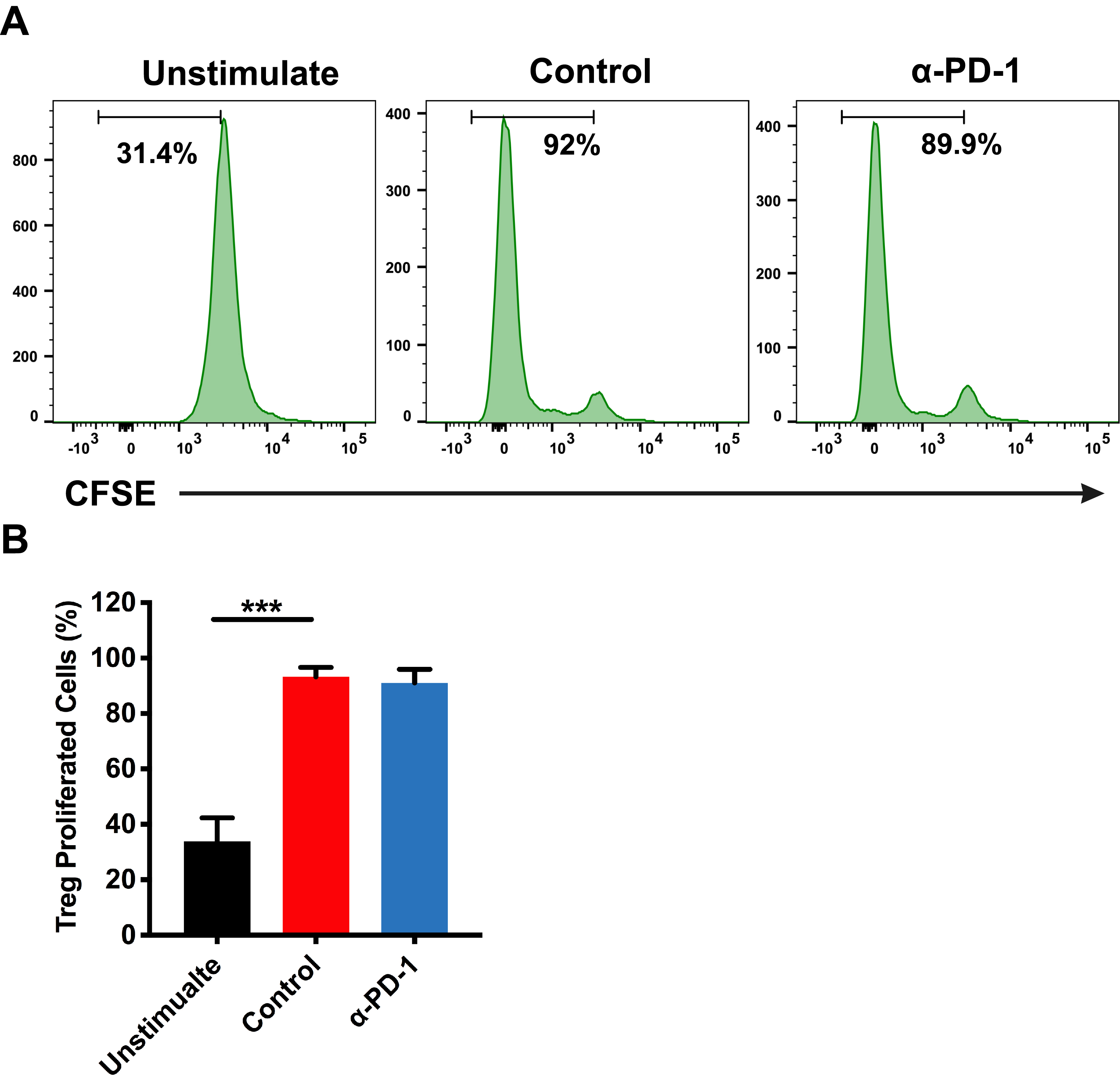

Supplement: Supplementary Figure 3 — The proliferation of Treg cells after different treatments. (A) Representative histogram of CFSE-labeled human peripheral Treg cells after different treatment (unstimulated, stimulated, and anti-PD-1). (B) Proportion of proliferation cells are shown (n=4; p < 0.05; p < 0.0001). Significance was calculated with one-way ANOVA, and all data are presented as mean ± SEM. [file Image_3.jpg]

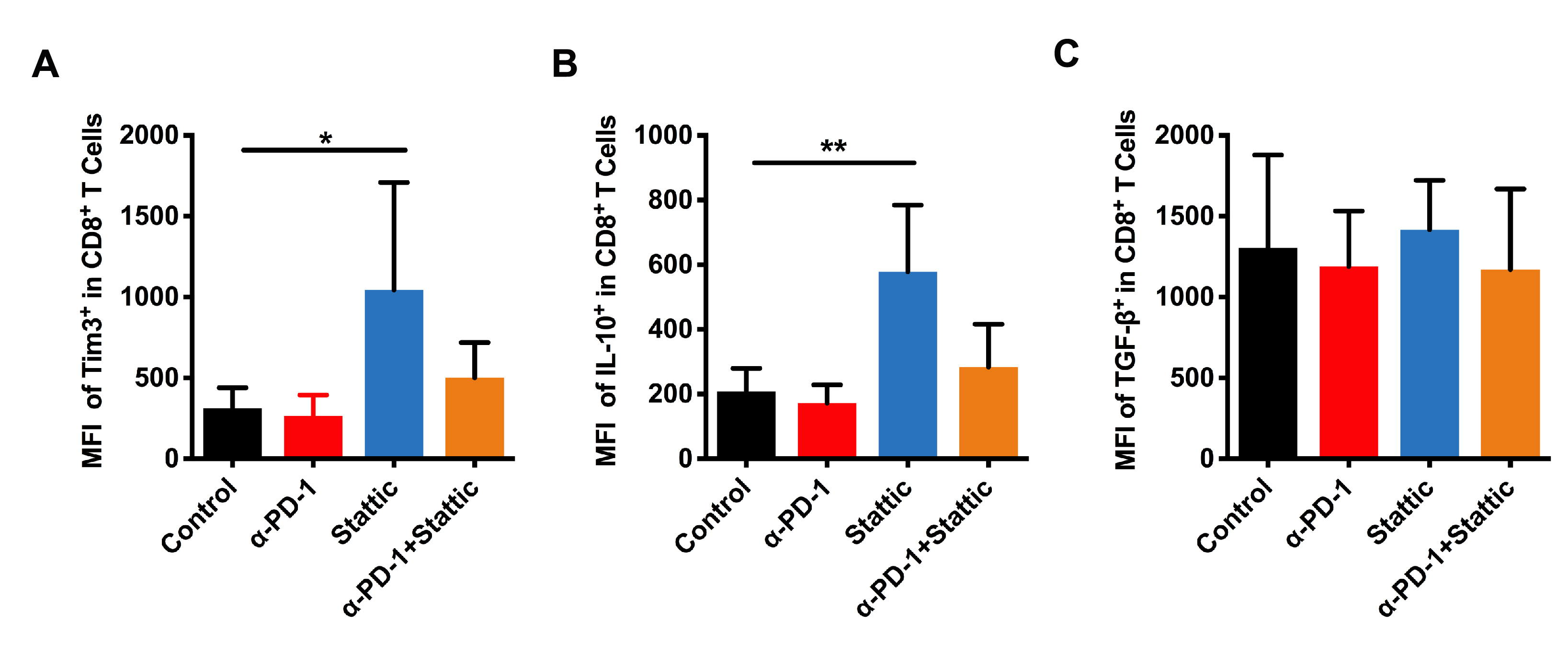

Supplement: Supplementary Figure 4 — Analysis of intratumoral CD8+ T cells and the associated phenotype and chemokine profile in vivo. (A-C) MFI of Tim-3 (A), IL-10 (B), and TGF-β (C) determined in CD8+ T cells was assessed by flow cytometry. Two-way analysis was performed to assess statistical significance between different groups (n=5; p < 0.05; p< 0.01). [file Image_4.jpg]
